# Examining how Ethics in Relation to Health Technology is Described in the Research Literature: Scoping Review

**DOI:** 10.2196/38745

**Published:** 2022-08-15

**Authors:** Emilie Steerling, Rebecca Houston, Luke J Gietzen, Sarah J Ogilvie, Hans-Peter de Ruiter, Jens M Nygren

**Affiliations:** 1 School of Health and Welfare Halmstad University Halmstad Sweden; 2 College of Allied Health and Nursing Minnesota State University Mankato, MN United States

**Keywords:** ethics, ethical principles, ethical theory, health technology, eHealth, digital health, telehealth, mHealth, health care, scoping review

## Abstract

**Background:**

Given the increased use of technology in health care, both in extent and application, the importance of understanding the ethical implications of new health technologies increases. Profound insight into the possible ethical implications of new health technologies enhances the research and development of such technologies and the likelihood of eventual successful implementation in clinical practice.

**Objective:**

This study aimed to gain an understanding of how and if researchers focused on health technologies describe the actual or possible ethical aspects of their research findings.

**Methods:**

An established framework for scoping reviews was used to guide the methodology. Studies published in PubMed over the last 10 years were included if they study or refer to ethics in relation to health technology as defined by established frameworks. In total, 14,532 articles were screened, 692 were retained for full-text evaluation, and 227 were included for data extraction.

**Results:**

In total, 250 (80.9%, N=309) studies were conducted in North America and Europe; literature review studies were dominant. Most studies (52.9%, 120/227) had no direct reference to any of the 4 basic ethical principles: beneficence, nonmaleficence, autonomy, and justice. In cases where studies referenced ethical theory, consequentialism dominated.

**Conclusions:**

When research about technology and ethics is published, the predominant focus is on its intent rather than its actual effect on patients. This lack of insight is problematic considering the vast advancement of technology in which ethics cannot keep up with understanding and offer insights on addressing ethical issues. This finding has implications for practice, research, and education.

## Introduction

Health technology is increasingly being used in various areas of health care. It provides ample options to meet societal needs in improving quality, optimizing resource use, and the coproduction of care within the health care system [1]. As health technology has moved beyond supporting the treatment of life-threatening or congenital diseases and into genomics, diagnosis, surveillance and big data, and artificial intelligence, the central ethical questions have shifted to issues around integrity and equity on both individual and system levels [2]. Such issues are concerned with challenges relating to the risk that technology is biased, builds on or even reinforces inequalities, and overturns the principles for how care has traditionally been practiced and the logical setup for structuring the care system [3].

Health technology has traditionally been regarded as neutral [4], and bioethics is dominated by developer perspectives on the application in clinical practice [5]. However, health technology is increasingly spanning beyond the traditional organization of health care; use in contexts outside of the health care organization, such as in-home environments and workplaces; and the active role of the patient in operating and providing the functionality of the technology [6]. This overturns the traditional relationship between physicians and patients as an essential component of current health care practice [7]. Furthermore, technology’s mediation of human-to-human relations may result in ethical dilemmas [8]. Therefore, a broader perspective is needed where both the context and future implications for human life are considered [9].

Given the increased use of technology in health care, both in extent and application, the importance of understanding the ethical implications of new health technologies increases [2]. Profound insight into the possible ethical implications of new health technologies enhances the research and development of such technologies and the likelihood of eventual successful implementation in clinical practice [10]. Due to the rate at which technology advances, bioethics is falling further behind in staying current with new, evolving ethical issues [11]. This is mainly due to a tradition of retrospective approaches examining ethical issues [12,13]. To address and learn from such studies, we propose that researchers and developers of new health technologies integrate ethical analysis early on in the research and development process.

Much has been written, discussed, and taught about the importance of performing health research involving human subjects ethically and by strict guidelines [14-16]. In addition, medical and health journals no longer publish research that has not gone through an ethical review by an independent ethics board [17]. These ethical review boards limit their evaluation to the ethics of the study itself by reviewing issues such as informed consent, coercion, and risks or benefits to study participants. Research ethics and the role of the ethical review boards limit themselves exclusively to the ethical nature of research studies. They do not consider the possible ethical and unintended effects of the research findings after the study has been completed [18]. This study’s objective was to understand if and how researchers focused on health technologies describe the actual or potential ethical aspects of their research findings.

## Methods

The methodology for this scoping review followed the methodological framework put forward by Arksey and O’Malley [19] and a previously described protocol [20]. In total, 4 stages were applied to map current knowledge and identify gaps: (1) identification of relevant literature, (2) selection of studies, (3) charting of data, and (4) synthesizing results.

### Identifying Relevant Literature

To ensure the research team adequately explored a broad array of health-related aspects unbiased without using specific predetermined health-related search terms, all articles were located through Pubmed, the major database for biomedical literature in life science journals and digital books. PubMed was selected as the sole source due to the large number of interdisciplinary and mainly health-related publications located in this database and the extensive and time-consuming search strategy using individual terms of technologies based on a nomenclature rather than a single or few broad and less precise terms such as “health technology.” The database search was conducted from September to October 2020. The health technologies, which were used as primary search terms, were identified through the Global Medical Device Nomenclature [21] and the nomenclature used in the World Health Organization (WHO) [22] report “Human Resources for Medical Devices, the Role of Biomedical Engineers.” The list included devices and techniques used to manage health care delivery, which parallels the definitions of technology and technique [23]. Searches were conducted using 181 search terms for *technologies* AND ethic*, allowing any of the suffixes of *ethic* (ie, ethic[s], ethic[al], ethic[ally], ethic[ist], ethic[ism], and ethic[ality]) to be included in a search of titles and abstracts. A time delimiter was set to include only articles published in PubMed in the last 10 years (2010-2020) from the date of the searches. The reason for including only recent literature is that the research field in health technology is constantly and rapidly developing and that we intended for this study to be based on current research in the field. Upon completing each search, all records were saved to a shared file and imported into the Covidence software (Veritas Health Innovation) for further analysis.

### Selecting Studies

Only studies that specifically mentioned ethics concerning a health technology or technique were included. The screening was conducted in collaboration with all 6 authors. All authors participated in regular meetings to discuss the inclusion and exclusion criteria interpretations. In the initial screening, the titles and abstracts were independently examined by 2 authors, who independently determined whether the article appeared to meet the inclusion criteria. A third author was included to resolve the conflict if a disagreement was apparent.

Studies were excluded if they met the following exclusion criteria: ethics concerning human health was not mentioned; ethics concerning technology was not mentioned; the authors did not elaborate on ethics; the authors referred to research ethics only; the study was not in English; not a research article; not a peer-review article; not a retrievable article; duplicate article; or animal research ethics. After that, the studies were assessed for eligibility based on the full text. Again, 2 authors independently reviewed each study, and in cases of disagreement or uncertainties, conflicts were resolved by a third author. The Covidence software allowed several authors to code and analyze the same data set simultaneously. The average interrater reliability in screening both records and full-text articles was satisfactory (κ*=*0.23 and κ*=*0.52, respectively). The authors followed the PRISMA (Preferred Reporting Items for Systematic Reviews and Meta-Analyses) reporting guidelines for systematic reviews to report the screening process [24].

### Charting the Data

A data extraction form was designed in the Covidence software to chart critical items from the final set of studies that made it through the extensive and rigorous screening process. Data extracted using the questionnaire form included the bibliographic details of the study; geographical location where the studies were carried out; type of research methodology used; types of technologies investigated; aims of the study; and ethical principles and theory referred to in the study.

### Synthesizing Results

The intention of the scoping review was not to synthesize evidence or aggregate findings from different studies but to map their characteristics. A 3-step process collated and summarized the data and presented a narrative account of existing literature. Each included article’s data were independently extracted by 2 authors, and a third author confirmed a consensus of each article’s extracted data. First, data extraction forms were used to report descriptive numerical analysis of the studies’ extent, nature, and distribution. Second, the Bloom taxonomy of measurable verbs was used to classify the level of critical thinking in the studies as determined by the verbs used in the aims of the studies. The taxonomy comprises 6 categories describing learning progression, including knowledge, comprehension, application, analysis, synthesis, and evaluation [25] (Table 1). Third, the ethical analysis was based upon Beauchamp and Childress’ [26] 4 principles approach, which inform the ethical decision-making in health care, and the 3 ethical theories—consequentialism, deontology, and virtue. Finally, a template for the extraction was developed with brief descriptions of the concepts used: beneficence—the duty to do good; nonmaleficence—avoiding causing harm to a person; autonomy—respecting the individual’s right to decide for him/herself; justice—fairness and duty to protect human dignity; consequentialism—the consequences of the chosen action determine whether the action is right or wrong; deontology—there are allowed, forbidden actions (eg, Kantian duty-based ethics); and virtue ethics—the moral actor is in the center. The analysis was carried out by searching for references to the ethical principles in the studies first. Next, the description and discussion were assessed to see if the ethical theories were referred to.

**Table 1:** Bloom taxonomy.

Category	Description	Example verbs
Knowledge	Remember: remember basic concepts and facts	List, name, recall, record, relate, repeat, state, tell, and underline
Comprehension	Understand: explain ideas or concepts	Compare, describe, discuss, explain, express, identify, recognize, restate, tell, and translate
Application	Apply: use information in new situations	Apply, complete, construct, demonstrate, dramatize, employ, illustrate, interpret, operate, practice, schedule, and sketch
Analysis	Analyze: see connection and pattern	Analyze, appraise, categorize, compare, contrast, debate, diagram, differentiate, distinguish, examine, experiment, inspect, inventory, question, and test
Synthesis	Evaluate or assess: justify opinion and decision	Arrange, assemble, collect, combine, comply, compose, construct, create, design, devise, formulate, manage, organize, plan, prepare, propose, and setup
Evaluation	Create: create new or innovative	Appraise, argue, assess, choose, compare, conclude, estimate, interpret, judge, justify, measure, rate, revise, score, select, support, and value

## Results

A total of 18,166 potentially relevant articles were identified in PubMed using the a priori medical technologies and techniques outlined in the Global Medical Device Nomenclature and WHO. Of these articles, 3534 were removed by the Covidence software because they were identified as duplicates.

**Figure 1:**
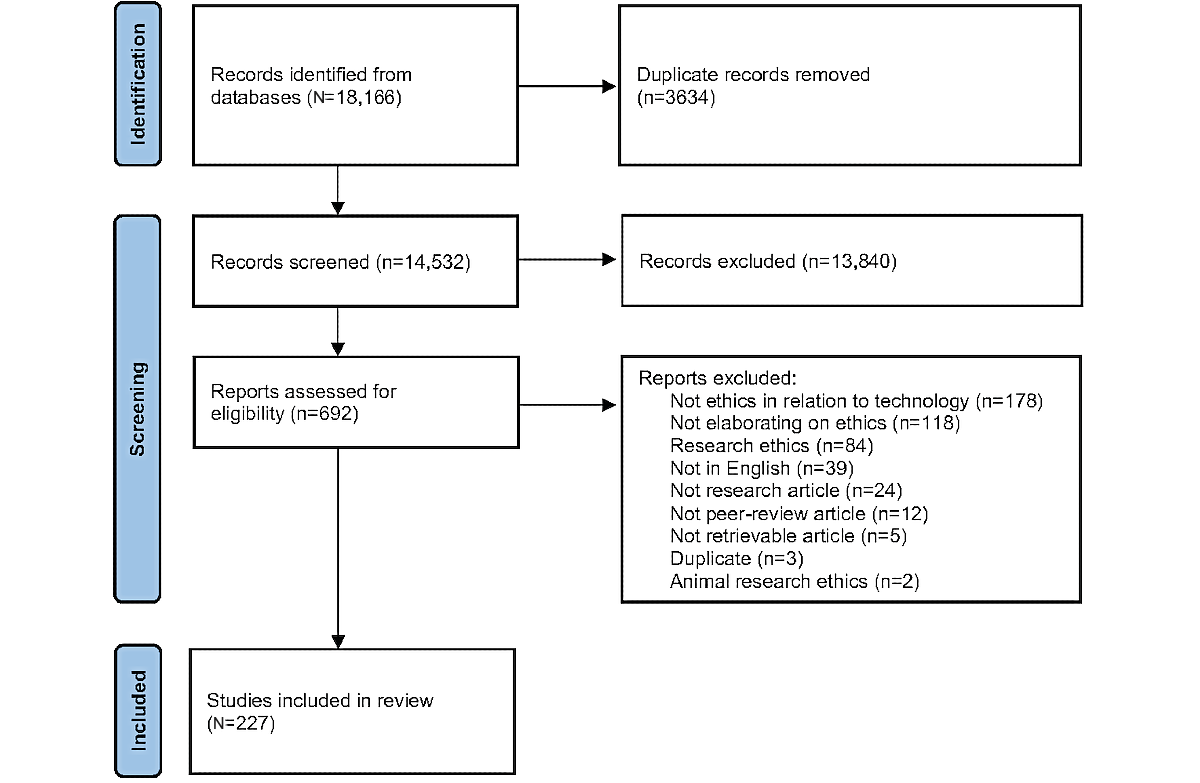
Flow diagram of the study selection process.

After the initial screening of titles and abstracts, 13,840 articles were excluded because they refer to non–technology-based ethics, non–health-related ethics, ethics concerning research methodology, or an ethical review board reviewed the research. The second-round screening of full-text articles included 692 articles and excluded 465 articles (Figure 1). The remaining 227 articles were deemed adequate for data extraction. A complete bibliographic list of included studies can be found in Multimedia Appendix 1.

The data extraction results indicated that 80.9% (250/309) of the articles originated from researchers from North America and Europe; empirical studies were also primarily based on data collected in these countries (Table 2). In addition, 81.9% (186/227) of the studies were literature and primarily narrative reviews with limited or no descriptions of the methodological approach of the review and little distinction between references to the literature versus the assumptions from the authors.

Of the empirical studies, qualitative studies dominated. Only 7 studies used pure quantitative and objective methodologies. None of the quantitative studies focused their research on developing or using validated measures for assessing ethics (Table 2). The articles were grouped based on the different health technologies or techniques central to the research. Based on the meaning units taken from the articles’ description of the respective technologies and techniques, 12 groups were initially formed and then merged into 6 groups describing the different types of technology areas with limited overlap between the areas (Table 2).

Of the 227 studies, 52.9% (n=120) had no direct reference to the 4 basic ethical principles: beneficence, nonmaleficence, autonomy, and justice. Among the 107 studies referring to 1 or more of the ethical principles, 244 references were made, of which 33.2% (n=81) only mentioned them in passing, without linking them to an ethical theory. The references referring to an ethical principle and an ethical reasoning around ethical theory (n=163) did so mainly in relation to consequentialism (69.3%, n=113), followed by deontology (26.4%, n=43) and virtue (4.3%, n=7; Table 3).

To understand the type of approach that the research on health technology and techniques had and thus provide an understanding of the conceptual level the technologies and techniques were studied and the connection to ethics that could be made, the formulations of the aims in the articles were studied. The specific aims of each article were examined to understand the research approach, which was used to determine the conceptual level of each study and provided clarity on the connection to ethics. Based on Bloom taxonomy, most studies (80.6%, 183/227) aim to use verbs at the conceptual levels of knowledge, comprehension, and analysis. In contrast, studies that investigated application, synthesis, and evaluation were sparse (Table 4).

**Table 2:** General characteristics of included articles (n=227).

Characteristic			Article, n (%)
**Country^a^ (n=309)**			
	Africa	6 (1.9)	
	Asia	17 (5.5)	
	Europe	127 (41.1)	
	North America	123 (39.8)	
	Oceania	30 (9.7)	
	South America	6 (1.9)	
**Research design (n=227)**			
	Narrative literature reviews	170 (74.9)	
	Qualitative	30 (13.2)	
	Systematic literature reviews	16 (7)	
	Quantitative	7 (3.1)	
	Mixed method	4 (1.8)	
**Technology and technique (n=227)**			
	Big data, information systems, and artificial intelligence	65 (28.6)	
	Technologies for treatment	58 (25.6)	
	Internet of things, eHealth, and mobile health	41 (18.1)	
	Gene editing and sequencing	28 (12.3)	
	Gene screening, testing, and diagnosis	23 (10.1)	
	Technologies for assistance	12 (5.3)	

^a^An article may have multiple countries of origin.

**Table 3:** Distribution of references to ethical principles and ethical reasoning around ethical theory (N=244).

Ethical principal	Virtue (n=7), n (%)	Deontology (n=43), n (%)	Consequentialism (n=113), n (%)	No references to ethical theory (n=81), n (%)
Beneficence	1 (14.3)	9 (20.9)	21 (18.6)	20 (24.7)
Nonmaleficence	1 (14.3)	5 (11.6)	18 (15.9)	17 (21)
Autonomy	4 (57.1)	18 (41.9)	56 (49.6)	22 (27.2)
Justice	1 (14.3)	11 (25.6)	18 (15.9)	22 (27.2)

**Table 4:** Distribution of verbs used in the study aims according to Bloom taxonomy.

Category, verb		Usage (N=227), n (%)
**Evaluation**		
	Assess	7 (3.1)
	Consider	3 (1.3)
	Compare	2 (0.9)
	Reflect	1 (0.4)
	Evaluate	1 (0.4)
**Synthesis**		
	Propose	4 (1.8)
	Categorize	1 (0.4)
	Point out	1 (0.4)
	Develop	1 (0.4)
	Speculate	1 (0.4)
**Analysis**		
	Explore	27 (11.9)
	Examine	20 (8.8)
	Analyse	7 (3.1)
	Investigate	6 (2.6)
	Focus	3 (1.3)
	Encourage	2 (0.9)
	Study	1 (0.4)
	Stimulate	1 (0.4)
	Inquire	1 (0.4)
	Inventory	1 (0.4)
**Application**		
	Present	10 (4.4)
	Offer	3 (1.3)
	Contribute	3 (1.3)
	Show	2 (0.9)
	Determine	2 (0.9)
	Assist	1 (0.4)
	Inform	1 (0.4)
**Comprehension**		
	Discuss	24 (10.6)
	Review	13 (5.7)
	Identify	13 (5.7)
	Highlight	8 (3.5)
	Understand	4 (1.8)
	Summarize	4 (1.8)
	Illuminate	3 (1.3)
	Give	2 (0.9)
	Classify	1 (0.4)
	Orient	1 (0.4)
**Knownledge**		
	Provide	14 (6.2)
	Address	11 (4.8)
	Outline	8 (3.5)
	Describe	4 (1.8)
	Introduce	1 (0.4)
	Underline	1 (0.4)
	Lay out	1 (0.4)
	Gather	1 (0.4)

## Discussion

### Principal Findings

Ethics and health care are closely intertwined, and it is not easy to discuss the moral principles presented in health care and the technologies used within its practice without considering the ethics involved. The findings show that more than 80% of the studies were conducted in North America and Europe and few in Africa and South America. These results are in line with research suggesting that although there is increasing research on digital health implementation in low- and middle-income countries, there is a lack of in-depth discussions of the ethical implications of health technologies in these settings [27]. The findings also show that more than 80% of the studies were literature reviews and that very few studies based their research on empirical data. In addition, primarily objective quantitative methodologies were sparse. These findings indicate the lack of discussion of the ethical issues concerning health technologies and that the limited discussions available have a poor grounding in empirical data. More then 50% of the included studies researched big data, information systems, artificial intelligence, and technologies used to provide various forms of treatment. This could reflect a tendency to raise more ethical concerns regarding implications within these technology areas instead of genetics and gene technology, gene-based screening, and technologies used for assistance during illness and rehabilitation. It could also reflect the current hype of applying health technologies based on health care data and artificial intelligence and that these technologies are more studied in general and with low reference to actual application and empirical grounding [28].

It is beneficial for the field of ethics in research and health care education to receive more measurable data in terms of the verbiage and principles surrounding ethics concerning health technology [2]. To understand how ethics have been analyzed, we looked at the usage and dichotomy of ethical principles in studying and discussing health technologies. Evaluating ethical principlism created a dynamic framework for addressing ethical dilemmas witnessed in medical practice and thus reflected in the literature [29]. The peer-reviewed research articles’ content was labeled using Beauchamp and Childress’ [26] 4 principles approach and the ethical theories describing virtue, deontology, and consequentialism. Either one, several, or none of the 4 principles were used to describe either or none of the 3 ethical theories. The findings in this study echo the concept that principlism itself does not set out a single consistent or coherent moral theory [29]. Therefore, viewing the dimensions and intricacies of ethics in patient care and clinical medicine is vital to understanding the complexity of ethics in health technology. The studies were further analyzed using Bloom taxonomy of measurable verbs [25]. Observing the arrangement of measurable verbs beginning with knowledge, the most basic level, and progressing through evaluation, the most complex level, provides valuable insight into the dimensions that ethics takes on in medical technology literature [30,31]. Understanding the broadness of the term ethics provided a greater scope behind how and why it is researched. Identifying ethics verbiage through this additional taxonomy enhances our understanding of the complexity of the research in which ethical issues are researched [31,32]. Unfortunately, the framing of the research, given the formulation of the research aims of the studies, had uneven conceptual distribution and was primarily of lower complexity.

Ethics informs education and professional standards, and changes in ethics are becoming increasingly apparent through the growing overlap between medicine and technology. The appreciation for the impact of ethics on the field of medicine increases as conversations about health technology application in health care continues to grow [31,33]. By summarizing the term “ethics” in health technology research, this study indirectly contributes to both health technology and ethics and informs practice and clinical decisions made every day [29]. In practice, it is evident that little work is being done proactively in identifying issues that might have unwanted or adverse results on patients. Thus, practitioners need to be trained and practice identifying and addressing ethical issues [34]. This practice needs to be a part of implementing and assessing new technologies as part of the evaluation process [35]. In addition, practitioners need to expand their curiosity beyond understanding what technologies do and understand what it does not do or does that is unwanted. Ethical committees and university centers for bioethics should be essential in helping practitioners obtain the needed skill set [36]. Examining the ethical and unintended effects as part of the research process must be required. Similar to addressing the study’s limitations and ensuring the following of ethical guidelines and regulations, the researchers should consider the possible unintended effects and ethical issues of their research regardless of the type of health technology [37]. This consideration will lead to a higher level of transparency and allow for guidance to practitioners as they implement and evaluate new technologies. Finally, regarding education, all researchers of new health technologies and techniques should have adequate training in ethics and how to evaluate unintended and ethical effects resulting from their work [36,37]. This thinking needs to transcend beyond the study’s research ethics review to evaluate the impact of their work. A study of whether a new technology is carried out ethically does not necessarily mean that the technology itself will not have ethical or undesirable effects. To integrate this as a standard in research on health technologies, supervisors and committees involved in doctoral education have a crucial role in ensuring that this education is an essential part of doctoral training to establish it as a natural skill set for the next generation of researchers, supervisors, and research leaders [38].

### Limitations

The selection of the sample in this study has both strengths and limitations. To avoid difficulties in defining the health relevance of different techniques and their application, the search for articles was limited to PubMed, the primary medical and health science research database. The combination of searching using keywords for ethics and technology based on WHO- and Global Medical Device Nomenclature–predetermined terminologies in this database gave a search result including records with high relevance to health technology in relation to human health. The strength of this approach is that the inclusion of studies concerning the technology’s relevance to human health was not limited based on search terms but based on screening a large number of records. The weakness of the approach is that research with the same focus but found in other databases was not included in the study. All screening and data extraction was done in pairs, and the definitions and interpretations of the inclusion and exclusion criteria were discussed in regular meetings. The study focused on ethics in relation to health technology in general, which gave an overview of how the field relates to issues regarding ethics. However, a more precise and applicable result would have been obtained if the study had been limited to a more precise technology area, such as big data and artificial intelligence. Due to limitations in the authors’ language skills, only articles written in English were sought, which is a limitation for the study.

### Conclusion

This study shows that research about technology and ethics predominantly focuses on intent rather than actual effect on patients. This lack of insight is problematic considering the vast advancement of technology in which ethics cannot keep up with understanding and offer insights on addressing ethical issues. A predominant focus on the ethical aspects of the direct consequences of health technology means that more difficult ethical considerations are overlooked, such as potentially unintended effects, researcher’s approaches to ethical issues, and expectations on abiding to regulations and standards. This finding identifies a need for a broader approach to ethical issues linked to health technology, something that should continue from the training of new researchers, in the design and funding of new research studies, and in the presentation and publication of new research results.

## Appendix

Multimedia Appendix 1 Complete bibliographic list of included articles.

## Abbreviations

PRISMA: Preferred Reporting Items for Systematic Reviews and Meta-Analyses
WHO: World Health Organization
